# Lagged acute respiratory outcomes among children related to ambient pollutant exposure in a high exposure setting in South Africa

**DOI:** 10.1097/EE9.0000000000000228

**Published:** 2022-11-07

**Authors:** Shumani Phaswana, Caradee Y Wright, Rebecca M Garland, Thulie N Khumalo, Rajen N Naidoo

**Affiliations:** a Discipline of Occupational and Environmental Health, University of KwaZulu-Natal, Durban, South Africa; b Environment and Health Research Unit, South African Medical Research Council, Pretoria, South Africa; c Department of Geography, Geoinformatics and Meteorology, University of Pretoria, Pretoria, South Africa; d Smart Places Cluster, Council for Scientific and Industrial Research, Pretoria, South Africa; e Department of Forestry, Fisheries and the Environment, Pretoria, South Africa

**Keywords:** Air pollution, Dose-response effects, Lagged effects, Lung function, respiratory symptoms, schoolchildren

## Abstract

Acute ambient air pollution impacts on the respiratory health of children may be lagged across time. We determined the short-term lagged effects of particulate matter (PM_2.5_), sulphur dioxide (SO_2_), and oxides of nitrogen (NO_x_) on the respiratory health of children living in low-income communities.

**Methods:**

A school-based study was conducted using a repeated measures design, across summer and winter, in four schools in each of four suburbs in the Vaal Triangle, South Africa. Data for PM_2.5_, NO_x_, and SO_2_ were obtained from monitoring stations within close proximity of the schools. Over 10 school days in each phase, grade 4 children completed a symptoms log and lung function tests. Parents completed a child respiratory questionnaire. Generalized estimation equations models adjusted for covariates of interest in relation to lung function outcomes and air pollutants including lag effects of 1–5 days.

**Results:**

Daily PM_2.5_, NO_x_, and SO_2_ median concentration levels were frequently higher than international standards. Among the 280 child participants (mean age 9 years), the prevalence of symptoms based on probable asthma was 9.6%. There was a consistent increased pollutant-related risk for respiratory symptoms, except for NO_x_ and shortness of breath. Lung function, associated with pollutant fluctuations across the different lags, was most pronounced for peak expiratory flow rate (PEFR) for PM_2.5_ and SO_2_. A preceding 5-day average SO_2_ exposure had the largest loss (7.5 L/minute) in PEFR.

**Conclusions:**

Lagged declines in daily lung function and increased odds of having respiratory symptoms were related to increases in PM_2.5_ and SO_2_ among a school-based sample of children.

**What this study adds**

This, one of the few such studies from sub-Saharan Africa, focuses on four very low socio-economic communities in a region legally designated as a high pollution area in South Africa, known as the “Vaal Triangle”. The study provides critical evidence of exposure-related lagged respiratory effects – both acute symptoms and acute changes in lung function among a cross section of schoolchildren selected without consideration to their health status. The repeated measures design captures acute effects within the sample over the summer and winter seasons. In this sample, acute dose-related effects at pollutant levels below the WHO Air Quality Guidelines were observed.

## Introduction

There is strong evidence for ambient air pollution affecting child respiratory health, worsening asthma, and being associated with chronic bronchitis, hospital admissions, and healthcare facility visits for upper respiratory infections or lower respiratory illnesses.^1–3^ Symptoms of asthma are triggered by increased ambient air pollution levels.^4–6^ The increasing prevalence of childhood asthma globally has focused attention on environmental pollution as a cause of increased respiratory morbidity.^7^

Compared with the earlier studies, particularly those with cross-sectional designs, recent epidemiologic studies have provided evidence for lagged effects of exposure among children.^1,8,9^ Understanding the lag response provides an opportunity for more appropriate interventions to protect vulnerable individuals. However, the duration of the lag and the intensity of the dose-response effect across these lags vary across studies. The delay in lagged exposure effects across studies ranges from day 0 to 12 days, whereas others report an average exposure of the days preceding the effect. Among more than 330 000 pediatric respiratory outpatient visits in Lanzhou, China, a 5-day lag represented the highest increase in visits for sulphur dioxide and nitrogen dioxide,^3^ although a 4-year study of pediatric outpatient visits across four Chinese cities, showed the largest particulate matter of 2.5-micron diameter (PM_2.5_) related-effects of single day lags at lags 0 and 1 and cumulative exposure at lag 7.^1^ Among the longest lagged effect reported among those 18 years and younger, was a cumulative 14 day, PM_2.5_-related respiratory outpatient visits.^10^ Although similar lagged responses are reported in adult cohorts,^11^ mortality across all ages seems to have a longer lagged effect – a South Korean study reported an almost 45-day lag on respiratory mortality associated with PM_10_.^12^ These inconsistencies may be related to the specific outcomes, pollutant types, exposure characterization or the selection of the populations under study.

Understanding exposure-related effects across different child subpopulations are critical. Although there is consistency in the literature for pollutant-related childhood respiratory outcomes, these are generally among samples of children with preexisting diseases, particularly asthma,^4–6^ or studies of outpatient visits and hospitalizations.^1,11^ Acute pollutant-related effects among randomly selected community-based samples of schoolchildren are limited.^13–16^ Although the estimates of effect are lower than that seen in studies among symptomatic or asthmatic children, these studies nevertheless consistently show increased risks for respiratory symptoms or lung function.

Few Southern African studies have reported air pollution-associated respiratory outcomes. Prevalence data from a study of children from seven schools in Durban, South Africa, the adjusted odds ratio (OR) comparing children in the industrially-intense south versus the less industrialized north of the city was 1.33 for doctor-diagnosed asthma. Similarly, the risk of children from the industry dense south having chronic symptoms of persistent asthma and airway hyper-reactivity compared with those from the north of the city was 1.14 and 2.49, respectively. There was also a two-fold increased risk for airway hyper-reactivity with SO_2_ exposure for children in the south.^17^

This study, conducted among four communities in an area legally designated as an air pollution priority area, (the “Vaal Triangle”), is in close proximity to petrochemical industries and coal-fired power stations, developed during the Apartheid era of town planning. Air pollution in the area regularly exceeds South African National Ambient Air Quality Standards for several criteria pollutants.^18^ The Vaal Air Pollution Study, conducted in 1990, among 14,053 children aged 8–12 years, described a substantially elevated prevalence of upper respiratory tract outcomes of sinusitis, hay fever, and earache of 44.3%, 26.3%, and 57.5%, respectively.^19^ Despite increased environmental regulation, exposure levels remain high in the Vaal Triangle.^18^

Our objective was to determine the relationship between acute childhood respiratory symptoms and acute lung function changes with daily and lagged fluctuations of PM_2.5_, SO_2_, and NO_x_ in this community-based sample of schoolchildren, in a designated pollutant priority area.

## Materials and methods

### Study design and population

**Figure 1.**
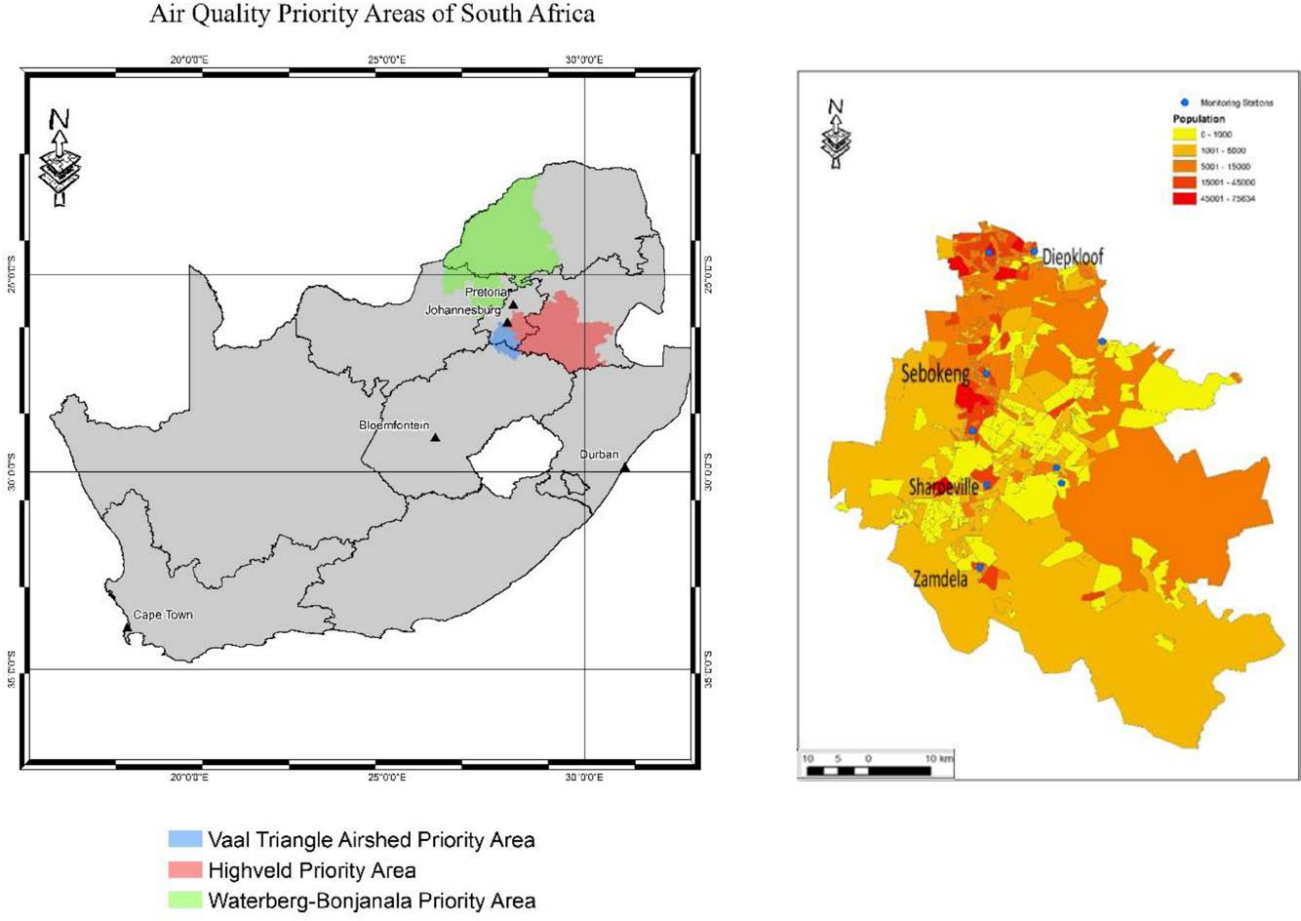
(A) Map showing the three Priority Areas in South Africa with the Vaal Triangle Airshed Priority Area shown in blue and (B) an enlarged map of the Vaal Triangle study area, showing the communities included in the study, the location of the monitoring stations and population density.

An observational study using a repeated measures design in a sample of schoolchildren was applied. The study was conducted in two phases in the years 2015/16 to reflect the austral summer (phase 1) and winter (phase 2) seasons. Four communities in the Vaal Triangle Airshed Priority Area (VTAPA) participated in the study: Diepkloof, Sharpeville, Zamdela, and Sebokeng and within these communities, one primary school per community was selected (Fig. 1). VTAPA was designated as a priority area under National Environmental Management: Air Quality Act 2004 (Act No. 39 of 2004) in 2006.^20^

Schools were identified based on the location of existing air pollution monitoring stations (Fig. 1A) overseen by the South African Weather Services. Using a database of schools in the area, together with Google Earth, schools within a three kilometer radius of the stations were shortlisted. These schools were then visited by the research team to determine whether they met the selection criteria, which included pupil population size (n ≥100 per Grade), presence and number of Grade 4 classes (n ≥1), and the extent of “bussing-in” of pupils from communities a distance away from the school or monitoring station. This was to ensure a minimum number of children living outside the range of the monitoring stations were included in the study. Of the nine schools identified and assessed, four schools, one in each area of interest, were selected. Each school had between three to four Grade 4 classes. The researchers randomly selected two Grade 4 classes in each of the schools for participation in the study, and all pupils in these selected classes were invited to participate in the study. Grade 4 was selected as these children were old enough to understand the instructions given for the test procedures.

### Air pollution exposure

Data of the environmental pollutants PM_2.5_, NO_x_, and SO_2_ for the two phases commencing 5 days before the start of the phase, were obtained from the South African Weather Services through South African Air Quality Information System (SAAQIS, http://saaqis.environment.gov.za/) for the four monitoring stations in the participating communities. The stations selected were regulatory monitoring stations whose instruments and methods follow reference methods as per South African legislation.^21^ The latter are based on international standards and best practices.^22,23^ For example, the PM2.5 reference method is the European standard EN 14907 method, and the SO_2_ method is based on the ISO 6767:1990.^23^ The pollutant databases were provided by SAAQIS as Microsoft Excel (Microsoft, Microsoft Excel, Redmond, Washington) spreadsheets for each of the monitoring stations and provided as 1-hour averages from the raw, continuously monitored data. The data provided were noted as quality controlled with details provided in their monthly reports.^24^ There were no data available from the Sebokeng monitoring station for the entire first phase. Data for all three pollutants were missing for the Sharpeville station for the period of 6 days in phase 2 and from the Zamdela station for half a day in phase 2.

### Child health assessment

Child health assessments were conducted during phases 1 and 2 to capture possible seasonal variations. During these phases, the children were required to complete a symptom and activity log twice a day (at approximately 07:30 h and 13:00 h) on school weekdays. The log collected information on symptoms, including cough, tight chest, wheeze, runny nose, and sore throat, since arrival at school and during school hours. Symptoms from the daily symptoms and activity logs were captured as “yes” if the child had reported to have had that symptom for that particular day, irrespective of whether or not it was reported in the morning or afternoon, or both times.

School children from three schools (the school from Diepkloof was excluded because of the unavailability of peak flow meters) performed three consecutive maneuvres for peak expiratory flow rate (PEFR) and forced expiratory volume in one second (FEV_1_) immediately following the completion of the symptoms and activity log in each of two sessions per day. The serial PEFR and FEV_1_ were conducted using the Airwatch (iMetrikus, Carlsbad, California) brand air monitor. Each child was provided with their own unique device, clearly labeled with their full name and study identification number. The mean PEFR and mean FEV_1_ of all valid blows for each child from each session were used in the data analysis. Each maneuvre performed by the child was assessed for meeting acceptability criteria. These measures were additionally compared against that obtained during formal maximal expiratory maneuvres performed by a spirometric technician, in accordance with the American Thoracic Society guidelines for conducting spirometry.^26^ Those responses that exceeded 120% or below 30% of the technician-obtained data were rejected. Among those that met these criteria, the mean measurement for the day was used in the analysis.^25^

Field supervisors provided intensive individualized training to the children in proper completion of the symptoms and activity log and the proper performance technique of the peak flow maneuvres immediately before the commencement of phase 1 with refresher training before phase 2. These field supervisors were present during the testing times to directly observe the expiratory maneuvres to ensure proper technique.

### Caregiver interviews

Trained fieldworkers administered questionnaires to parents or primary caregivers of the participating children. Components of the questionnaire included demographic information and an assessment of the presence and severity of respiratory and other relevant symptoms using a standardized, validated questionnaire from the South Durban Health study.^17^ Validated questions included wheezing, coughing, chest tightness, shortness of breath, activity limitations, and potential risk factors such as exposure to cigarette smoke, biomass usage, pets at home, indoor home environment, and preexisting medical conditions. All questionnaires were available in English and Sotho, the local languages. Interviews were conducted in the language of choice of the respondent by an interviewer who was fluent in the language.

Children were classified as having “any asthma”, “mild intermittent asthma”, “persistent asthma”, or “moderate to severe asthma” based on their responses to the standardized respiratory symptoms questions from the United States National Asthma Education and Prevention Programme.^27^ These categories were used in the analysis and is detailed in the ematerial; http://links.lww.com/EE/A204.

### Statistical analysis

All data were analyzed in Stata (StataCorp, 2015, Stata Statistical Software: Release 14, College Station, TX, StataCorp, TX, StataCorp LP). There were several dependent variables, namely, mean daily FEV_1_ and PEFR; symptoms of cough, wheeze, shortness of breath, and chest tightness as reported in the daily symptoms and activity logs.

Because of the repeated measures design, and correlation of observations, Generalized Estimation Equations (GEE) models were used to adjust for the various covariates of interest, although recognizing the repeated participation of the children across each day and in the two phases (“phase” was included in the model, and served as a proxy for “season”). The GEE models were developed for categorical outcome variables such as the presence (yes/no) of specific respiratory symptoms (i.e., cough, wheeze, shortness of breath, and chest tightness). These were models adjusted for covariates, apriori known to be associated with the outcomes of interest, such as caregiver education (high school or tertiary), caregiver smoking status, socio-economic status, (annual income categories), exposure to environmental tobacco smoke (presence/absence) and household allergens (pets, pests, and stuffed toys) and source of energy (electricity, wood, coal, paraffin and gas; wood, and coal were considered as biomass exposure) and attending school. Similarly, GEE models were developed for the lung function outcomes, which were further adjusted for sex, age, and height.

The hourly average pollutant data that was available was converted into 24-hour averages. All pollutant data were log transformed before entry into the regression models. The models included lag effects of pollution exposure for the preceding 1–5 days (defined as Lag 1, Lag 2, Lag 3, Lag 4, and Lag 5), as well as a 5-day average. Both single lag effects and distributed lags were modeled.

Because of the interaction between pollutants, as well as the interaction between the different lags, we investigated whether multipollutant distributed lag models or single pollutant-single lag models better described out data. To achieve this, we first investigated the correlation between the various pollutants and also assessed the variance inflation factors (VIF) in the development of the models.

### Ethics approval and consent

Research ethics approval was granted by the Council for Scientific and Industrial Research Ethics Committee (Ref: 69/2013). The study protocol was also endorsed by the Biomedical Research Ethics Committee of the University of KwaZulu-Natal. Approval to conduct the study was obtained from the Gauteng and Free State Provincial Departments of Education. Permission from each of the school’s principals to conduct the study within the school premises was also obtained. Informed consent was obtained in writing from all the participants’ primary caregivers and informed assent was obtained from the participating students.

## Results

### Pollutant exposure

All three air pollutants, PM_2.5_, NO_x_, and SO_2_ had higher median levels in the winter period (phase 2) than the summer (phase 1) (Table 1). These measurements were averaged for over 19 days for three sites, with Sebokeng having no data for the summer phase. Similarly, during the winter phase, the three sites’ measurements were averaged for 19 days, except for Sharpeville which was averaged over 13 days owing to missing data. The daily median for PM_2.5_ across the sites ranged from 4.8 µg/m^3^ to 48.9 µg/m^3^ and 6.5 µg/m^3^ to 115.5 µg/m^3^ in summer and winter, respectively. The range in daily medians for NO_x_ across sites was 3.4 ppb to 67.1 ppb in summer and 5.5 ppb to 123.7 ppb in winter. For summer, the daily median range in SO_2_ concentration was 0.6 ppb to 58.2 ppb and 0.8 to 21.8 ppb in winter. The highest PM_2.5_ and NO_x_ levels were observed at the Sharpeville air monitoring station during the winter, although the highest SO_2_ level was observed at the Zamdela air monitoring station during the summer.

**Table 1.** 24-hour median and range in outdoor concentrations for SO_2_, NO_x_, and PM_2.5_ at the four study sites for summer and winter phases

Pollutant		Air monitoring sites			
		Diepkloof (school 1)	Zamdela (school 2):	Sharpeville (school 3)	Sebokeng (school 4)
Phase 1 (summer)					
PM_2.5_ (µg/m^3^)	n	392	456	495	N/A
	Median	17.35	17.06	22.00	
	IQR	10.08–23.43	8.40–26.68	13.03–37.11	
	Range	0.44–85.59	1.31–210.92	3.33–101.2	
NO_X_ (ppb)	n	398	456	495	N/A
	Median	26.29	13.59	16.54	
	IQR	16.41–42.68	6.67–22.43	1.009–26.64	
	Range	5.99–300.34	1.00–90.78	2.62–98.47	
SO_2_ (ppb)	n	396	456	495	N/A
	Median	1.40	2.27	2.56	
	IQR	1.08–2.37	0.72–11.78	1.73–7.01	
	Range	0.71–22.05	0.44–103.38	0.94–49.39	
Phase 2 (winter)					
PM_2.5_ (µg/m^3^)	n	453	444	329	453
	Median	22.28	25.69	50.22	28.87
	IQR	14.8–30.74	13.50–49.63	20.00–77.40	18.33–45.1
	Range	0.41–220.31	1.20–281.98	3.15–251.32	0.89–349.14
NO_X_ (ppb)	n	453	221	305	453
	Median	36.47	11.46	43.58	20.62
	IQR	22.42–70.88	4.05–25.89	20.40–105.08	8.79–47.28
	Range	7.73–401.11	0.02–209.50	8.24–338.23	1.81–207.97
SO_2_ (ppb)	n	453	444	336	453
	Median	5.02	2.99	4.77	2.88
	IQR	3.38–7.43	1.36–7.15	30.3–7.82	1.59–5.59
	Range	1.23–105.5	0.58–115.98	1.48–62.07	0.13–69.79

N/A indicates not available.

### Child demographics

The mean age of the 280 school children participating in the study was 9.3 years (SD: 0.7), with 52% being female. Only a small percentage of primary caregivers had tertiary education (12%). Forty-one percent of households reported a presence of environmental tobacco smoke, with 29.3% having a smoker in the household (Table 2).

**Table 2.** Demographics, household characteristics and child respiratory outcomes as reported by the parent/primary caregiver of the child (n = 280)

Child, parent/primary caregiver and household characteristics	Response (n [%])(unless indicated otherwise)
Child age, mean (years) (SD)	9.3 (0.7)
% Female	146 (51.7)
Caregiver education level	
No schooling	4 (1.4)
Grade 1–6	47 (16.70)
Grade 7–9	98 (34.6)
Completed high school	100 (35.1)
Tertiary	31 (12.0)
Smokers in the household	82 (29.3)
Primary caregiver smokes	25 (8.9)
Any environmental tobacco smoke exposure	116 (41.4)
Biomass fuel exposure	51 (18.2)
Child respiratory outcomes	
Doctor-diagnosed asthma	10 (3.6)
% with current asthma	6 (60.0)
Ever treated for asthma	9 (90.0)
Chronic bronchitis	8 (2.8)
Hay fever	5 (1.8)
Chronic cough	13 (4.6)
Chronic phlegm	16 (5.7)
Shortness of breath	14 (4.9)
Ever sound wheezy	17 (6.0)
2 or more of such episodes	3 (17.6)
Required treatment for attacks	3 (17.6)
Breathing normal between attacks	2 (11.7)
Hospitalized for wheezing	3 (17.6)
Parent/primary caregiver report of asthma-like symptoms severity	9 (3.2)
Moderate to severe	8 (2.8)
Mild persistent	10 (3.6)
Mild intermittent	255 (90.4)
None	

### Child respiratory health outcomes

The prevalence of reported doctor-diagnosed diseases and respiratory symptoms was low, generally not exceeding 6% (Table 2). Doctor-diagnosed asthma had a prevalence of 3.6%. However, when asthma was classified according to responses to standardized respiratory symptoms questions, the prevalence of “any” asthma was 9.6%.^27^ The diagnosis of asthma for all children was made before the age of 5 years.

### Symptoms and lung function recorded during phase 1 (summer) and phase 2 (winter)

The most commonly reported respiratory symptoms during phase 1 were cough and runny or blocked nose (Table 3). The overall prevalence of respiratory symptoms was higher during the winter phase (phase 2) compared with phase 1. Wheezing and symptoms of the runny nose was found to be statistically significant (*P* < 0.05) across the seasons, with a higher prevalence in winter.

**Table 3.** The prevalence^a^ of daily symptoms recorded during the two phases

	Daily reporting of symptoms summer (phase 1)		Daily reporting of symptoms winter (phase 2)	
Symptom	(n)^c^	(%)	(n)^c^	(%)
Cough	1,419	56.3	1,564	56.3
Wheeze^b^	711	28.3	872	31.3
Tight chest	689	27.5	730	26.3
Shortness of breath	656	26.1	688	24.7
Headache	966	38.4	1,003	36.0
Sore throat	772	30.7	885	31.8
Runny or stuffy nose^b^	1,047	41.7	1,312	47.1
Watery/burning eyes	968	38.5	1,030	36.9

^a^Prevalence based on presence of symptom per day per child in each phase. The maximum total person-days = 280 children × 10 days, but not all children participated every day, hence a varying denominator.

^b^Statistically significant difference in prevalence across seasons (*P*-value <0.05).

^c^The n reflects the number of positive responses for that symptom over the potential 2800 participant-days.

The handheld device recorded overall mean FEV_1_ was similar across phases (1.6l [SD: 0.5]), with PEFR in phase 2 (259.6l/min [SD: 60.3]) statistically significantly (*P* < 0.05) higher compared with that in phase 1 (252.8l/min [SD: 59.3]) (data not shown)

### Association between air pollutants and respiratory symptoms

The exposure measures exhibited multicollinearity in two dimensions: (1) within exposure across lags; and (2) within lags across pollutants. The VIFs, when including all lags into single pollutant models was 4, 7.5, and 22 for NOx, SO_2_, and PM_2.5_, respectively. Similarly, the increase in the variance of estimates when all three pollutants were in the model ranged from 2.2 to 3.5. This evidence of multicollinearity made distributed lag models and multiexposure models unstable. For this reason, only the single-exposure/single lag models are presented here.

**Figure 2.**
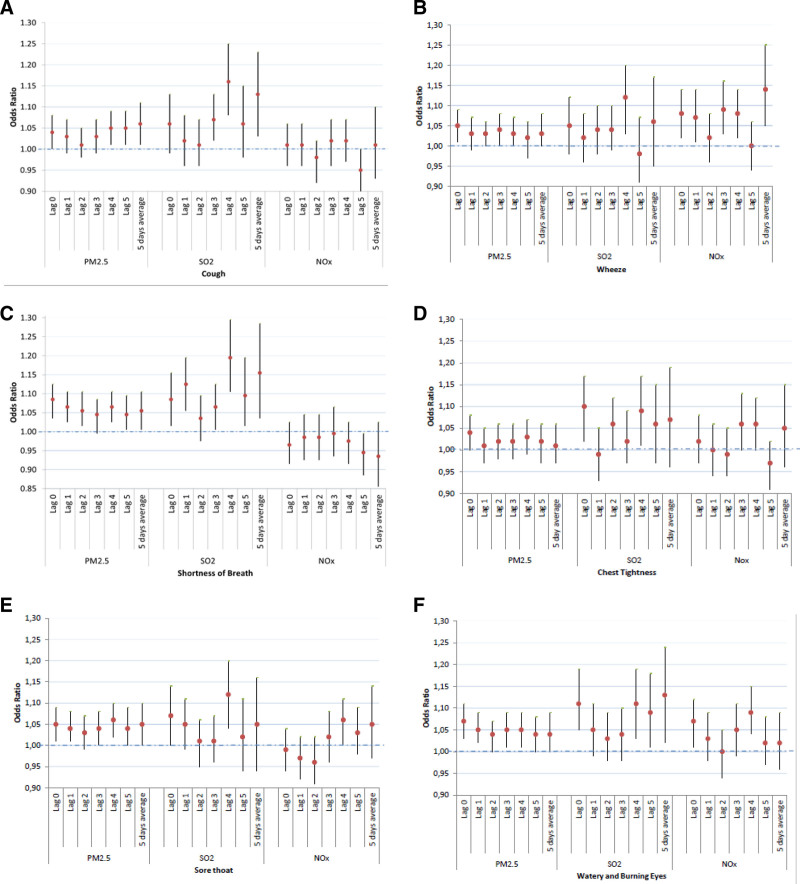
Association (as odds ratios depicted by red dot) and 95% confidence intervals (depicted by tails) between pollutants (per unit increase in interquartile range) and symptoms of (A) cough; (B) wheeze; (C) shortness of breath; (D) chest tightness; (E) sore throat; and (F) watery and burning eyes from GEE regression models adjusting for age, sex, environmental tobacco smoke, caregivers’ education level and biomass fuel usage. GEE, Generalized Estimation Equations.

The number of observations included in each of the pollutant/symptoms models ranged from 2600 through to over 4000. Although confidence intervals included the null effect, there were consistently increased pollutant-related risk estimates for all of the respiratory symptoms, except for that of NO_x_ and shortness of breath (Fig. 2). Similarly, the nonstatistically significant increased OR were evident across most of the lags investigated, as well as when investigating a five-day average exposure. There were consistently statistically significant relationships seen in the Lag 4 across all pollutants, although for the 5-day average, estimates were significant generally. However, they were uniformaly not significant for NOx exposure and chest tightness and for SO_2_ exposure-related wheeze. Apart from chest tightness, the relationship between PM_2.5_ and symptoms was most consistently statistically significant when compared with the other pollutants, across all lags, and with narrower confidence intervals not including the null effect. The ORs for PM_2.5_ for the various symptoms were located within a narrow range (1.01–1.07). Although the ORs for SO_2_ were also greater than 1 for most outcomes investigated, the confidence intervals were much wider (Fig. 2).

Unlike PM_2.5_, the range of ORs per increase in interquartile range (IQR) of SO_2_ was between 1.01–1.2, suggesting that the effect of SO_2_ exposure may be greater than that of the other pollutants, but with less stable effect estimates. The effect of NOx on symptoms was inconsistent: there were generally estimates of increased risk for wheeze and watery, burning eyes, but absent for shortness of breath, although for chest tightness and sore throat effects were seen in the later lags. However, these results for NOx must be interpreted with caution given the large confidence intervals, often including estimates of no effect.

### Association between air pollutants and lung function

**Figure 3.**
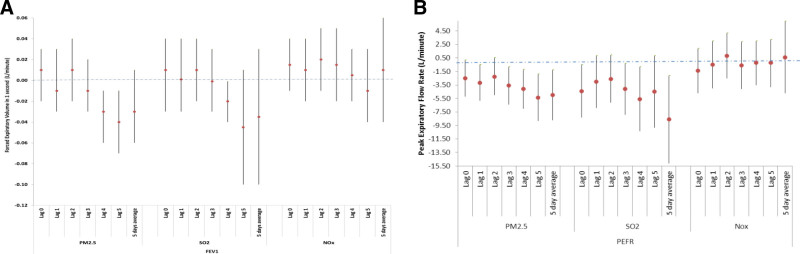
Daily changes (red dot depicts the point estimate and tails depict the confidence intervals) in (A) FEV_1_ and (B) PEFR in association with the IQR of daily pollutant levels for current day exposure and Lags 1 to 5 days and 5-day average, adjusted for age, height, sex, environmental tobacco smoke, caregivers’ education level and biomass fuel usage. FEV_1,_ forced expiratory volume in one second; PEFR, peak expiratory flow rate.

The models investigated daily changes in lung function parameters (FEV_1_ and PEFR) associated with either current day (Lag 0), preceding days one to five (Lag 1 to Lag 5), and the preceding 5-day average pollutant-related declines in lung function per IQR (Fig. 3). In these lung function/pollutant models, observations ranged from just over 1000 through to 1600, reflecting the absence of one of the participating schools in this component of the study. Although not always statistically significant, the pollutant-related decline was most pronounced for PEFR for PM_2.5_ and SO_2_ and was inconsistent for NOx. PM_2.5_ was associated with losses in FEV_1_ ranging from 0.01 to 0.04 L and a 2 to 5.5 L loss in PEFR, although the losses associated with SO_2_ ranged from 0.035 to 0.05 L in FEV_1_ and 2–8 L in PEFR. The largest pollutant-related statistically significant loss in lung function was demonstrated for PEFR and the preceding 5-day average exposure of SO_2_. For both these pollutants, the PEFR effects were more consistent in the expected direction than that seen for FEV_1_, and generally statistically significant. The effects noted with NOx for both outcomes always had confidence intervals including the null effect.

## Discussion

Our repeated measures study of 280 primary school children in a designated high pollution region, showed important pollutant-related effects for acute respiratory outcomes. There were findings of increased symptoms and lung function changes following short-term exposure ranging from lag 0 (current day) through to a 5-day lag, as well as a 5-day average. The findings were generally in the expected direction for two of the three pollutants under study: PM_2.5_ and SO_2_, particularly in the later lags, although these were sometimes not statistically significant. The pollutant-related increased risk was seen for outcomes of cough, wheeze, shortness of breath, watery and burning eyes, and sore throat and PEFR, particularly with PM_2.5_ exposure. Although not consistently statistically significant and less robust, the effects were greater with exposure to SO_2_.

Findings of lagged respiratory effects associated with pollutant exposure provides a better understanding of the exposure-response relationship. Although effects are likely to be airway irritant induced, particularly from larger particles, they may also be as a result of immunological and biochemical reactions. This has been postulated for oxides of nitrogen.^28^ PM_2.5_ and SO_2_ may have a more irritant action, and delayed responses are possible. Understanding the lagged effects may influence appropriate interventions among exposed children, such as individual clinical management and health services preparation.

Our findings of increased ORs for the various symptoms across the various lags within a narrow range of 1.01–1.2 is in keeping with that reported in other studies.^29,30^ A previous South African study reported similar ORs from single pollutant models per increase in the IQR of the criteria pollutants.^31^ In the latter study, there were small differences in the various lags examined, or in the 5-day averages. The variations seen across studies and across lags may be related to differences in the selection of the panels, with some including both asthmatics and nonasthmatics, and most focusing on asthmatic children only. These findings are not always consistent, as reported from the study on rural Japanese schoolchildren and sandstone exposure, where PM_2.5_ and suspended PM were not associated with an increased risk.^32^

Our lung function-pollutant relationship findings were inconsistent for the various pollutants. The FEV_1_ effects were most pronounced for the advanced lags (lag 3 to lag 5) for PM_2.5_ and SO_2_, this was statistically significant only for Lags 4 and 5. The largest statistically significant loss in lung function for preceding 5-day average exposure was seen for SO_2_. These lung function-related lagged effects have been reported in a variety of settings, including random community-based samples, such as our study.^15,31^ The findings are more consistent among studies of asthmatic children.^33–35^ The findings for PEFR showed greater consistency, in the expected direction, and were generally significant for PM and SO_2_. The effects for NOx showed no decline in PEFR. The responses to NO_x_ have varied across studies, with some showing distinct dose-response effects.^5,36^ A meta-analysis, however, showed no associated decline with NO_2_.^37^ The differences seen between the two measures of lung function are probably owing to a testing effect within the cohort: the effort required by FEV_1_ maneuvre is technically more demanding than the PEFR requirements, particularly among children. Even though trained fieldworkers observed the efforts of the schoolchildren in their classrooms, this was not performed on an individual basis.

In the analysis of our data, we considered both single and multipollutant models. However, because of the substantial correlation between pollutants, we could not consider multipollutant models. Similarly, we investigated distributed lag modeling including multiple lags within the model. Despite the advantages from such modeling, the multicollinearity that was evident in our data suggested that single pollutant, single lag models were the most appropriate.

Levels of air pollutants PM_2.5_, NO_x_, and SO_2_ were higher during the winter sampling period compared with the summer period. This may be a result of winter inversion layers or because of the increase in biomass burning as a means of home heating in the colder months. The prevalence of self-reported child respiratory symptoms was similarly increased. The reported chronic symptoms of cough, wheeze, and phlegm among the children were low, however, using more sensitive standardized classifications of probable asthma, “symptoms–defined any asthma” was 9.7%. Increased risk for increasing exposure to the different pollutants was seen consistently for the symptoms of wheeze for all pollutants and for chest tightness and cough with PM_2.5_ and SO_2_ exposure.

The levels of pollution documented in each of the communities during the period of study, were at modest levels when compared with other reports in the literature. According to the World Health Organization (WHO) reports annual mean levels of PM_2.5_ range from 1.6 µg/m^3^ to 20 µg/m^3^ for the advanced economies and findings of above 100 µg/m^3^ to 217 µg/m^3^ from developing countries.^38^ Similarly SO_2_ and NOx annual mean levels in the ranges of <5ppb to 70ppb have been reported in Europe and other developed countries, positioning the pollutants in our study at the upper end of this range, particularly for NOx.^39,40^

The communities under study form part of the Vaal Triangle Airshed Priority Area, as defined by the national government.^20^ The region consists of multiple point sources of pollution, including large petrochemical plants, coal-based power stations, as well as residential pollution through the use of biomass fuels and controlled agricultural field burning. The South African National Ambient Air Quality Standards (NAAQS) for 1-hour averaged NO_2_ is 106 ppb and SO_2_ is 134 ppb. The 24-hour averaged PM_2.5_ standard is 40 µg/m^3^. The monitoring stations used here are often out of compliance with annual NO_2_ standards, 24-hour and annual PM_2.5_ standards, although SO_2_ concentrations at these stations are not.

The study is characterized by several strengths including a high participation rate (89%); interviews with both participating child and parent/primary caregiver using trained interviewers with standardized, translated instruments; and spirometric assessments conducted by a trained and experienced technician. All aspects of the two phases were conducted following prior training of the participants and were performed under direct observation of trained supervisors. The study air pollution data, despite the missing data, were obtained under strict quality assurance and are subjected to detailed review and validation. The repeated measures study design that was employed allowed measurement of acute health changes in relation to daily fluctuations with air pollutants. The advantage of this design provides each participant as their own control over the course of the assessments, and this accounts for any unmeasured factor that does not vary on a daily basis.

Despite these strengths, there are several shortcomings that may have influenced the study findings. Monitoring exposure of each participant individually, or at each school, during the two phases would have been the ideal approach but was too costly. The researchers’ approach to use schools in close proximity to existing monitoring stations was the most economical, epidemiologically-acceptable method. Other important time-varying covariates are temperature and humidity. We did not have access to this data. However, day-to-day temperature and humidity fluctuations are not substantial in this region. Variations across the hot and cold months were adjusted by the multiple “phase” design. The response rate for spirometric assessments was 83.4%. This was largely because of children absent at school during the time of the assessment. These children are likely to have been those that were among the less healthy among the participants. However, we are not able to determine whether this created a bias in our results. In addition, although most children lived in close proximity to the school, children are likely to have been bussed in from surrounding areas. This will have influenced their exposure.

The study was conducted in 2015/16, however, the exposure levels experienced by these communities have not changed, and in some instances increased owing to the national energy demands from the coal-fired power stations in these areas. The socio-economic conditions, particularly health and health services in these communities have probably worsened following the COVID-19 pandemic, increasing the likelihood for adverse health outcomes, and hence strengthening the relevance of our findings.

Lung function decline in relation to PM_2.5_ and SO_2_ as well as consistent increased pollutant-related effects for all respiratory symptoms (except for some in relation to NO_x_) occurred in the context of relatively low ambient air pollution levels over the periods of study. Median air pollutant concentrations were typically below the NAAQS, with the exception of some exceedances (evident in the upper reading of the ranges, particularly for PM_2.5_ and NOx in all four sites during the winter phase (Table 1). The NAAQS are less stringent than either the 2005 or the recently announced 2021 World Health Organization Air Quality Guidelines.^41^ Thus in many instances, exposure levels exceed the global health-based standards. The lung function and respiratory symptoms variation in response to the short-term fluctuations in air pollution suggest that the South African National Air Quality Standards should be reconsidered to protect the respiratory health of vulnerable groups such as children, particularly those with asthma.

In our study, because of the lack of NO_2_ being monitored at all stations, we defaulted to the use of NO_x_ such that we could describe pollutant-outcome relationships across all the participating schools in a similar manner. To understand the likely effects of NO_2_, we determined the correlation between this pollutant and NO_x_ for those stations for which we had corresponding data. Correlations were moderate to moderately strong (R^2^ ranging from 0.53 to 0.72), suggesting that the fluctuations in respiratory outcomes will have followed a similar trend.

The findings of asthma defined based on responses to validated symptoms questions outcomes suggest that formal healthcare utilization may be inadequate in these communities. Strategies to strengthen health services and encourage health services utilization is necessary, given the prevalence of “symptoms-defined probable asthma”.

### Conclusion

In conclusion, our findings provide further evidence for pollutant-related declines in lung function among schoolchildren, which vary in statistical significance across pollutants and lags of exposure. Our findings are notable given that this was a community-based sample, consisting of both asthmatics and nonasthmatics.

## Conflicts of interest statement

The authors declare that they have no conflicts of interest with regard to the content of this report.

## Acknowledgements

The authors would like to acknowledge the participating schools, school principals, teachers, parents and schoolchildren. The authors would like to thank the South African National Department of Environmental Affairs for the funding of the study, the South African Weather Service, and SAAQIS for the air quality data. We express our thanks to Dr. Graciela Mentz (University of Michigan) for her assistance and advice for the statistical analysis.

## Supplementary Material



## References

[1] Li Y Li C Liu J. An association between PM(2.5) and pediatric respiratory outpatient visits in four Chinese cities. Chemosphere. 2021;280:130843. 34162098 10.1016/j.chemosphere.2021.130843

[2] Qiu H Chuang KJ Bai CH. Association of ambient ozone with pneumonia hospital admissions in Hong Kong and Taipei: a tale of two Southeast Asian cities. Environ Int. 2021;156:106634. 34015667 10.1016/j.envint.2021.106634

[3] Ma Y Yue L Liu J. Association of air pollution with outpatient visits for respiratory diseases of children in an ex-heavily polluted Northwestern city, China. BMC Public Health. 2020;20:816. 32487068 10.1186/s12889-020-08933-w PMC7265648

[4] Zhang Y Ding Z Xiang Q. Short-term effects of ambient PM(1) and PM(2.5) air pollution on hospital admission for respiratory diseases: Case-crossover evidence from Shenzhen, China. Int J Hyg Environ Health. 2020;224:113418. 31753527 10.1016/j.ijheh.2019.11.001

[5] Cisneros R Gharibi H Entwistle MR. Nitrogen dioxide and asthma emergency department visits in California, USA during cold season (November to February) of 2005 to 2015: a time-stratified case-crossover analysis. Sci Total Environ. 2021;754:142089. 33254941 10.1016/j.scitotenv.2020.142089

[6] Liu L Liu C Chen R. Associations of short-term exposure to air pollution and emergency department visits for pediatric asthma in Shanghai, China. Chemosphere. 2021;263:127856. 32822929 10.1016/j.chemosphere.2020.127856

[7] Dharmage SC Perret JL Custovic A. Epidemiology of asthma in children and adults. Front Pediatr. 2019;7:246. 31275909 10.3389/fped.2019.00246 PMC6591438

[8] Zhai G Zhang K Chai G. Lag effects of size-fractionated particulate matter pollution on outpatient visits for respiratory diseases in Lanzhou, China. Ann Agric Environ Med. 2021;28:131–141. 33775079 10.26444/aaem/132179

[9] Zhang H Liu S Dou Q. Association between ambient air pollutants and pneumonia in Wuhan, China, 2014–2017. Atmosphere. 2022;13:578.

[10] Chai G He H Sha Y. Effect of PM(2.5) on daily outpatient visits for respiratory diseases in Lanzhou, China. Sci Total Environ. 2019;649:1563–1572. 30308924 10.1016/j.scitotenv.2018.08.384

[11] Slama A Śliwczyński A Woźnica J. Impact of air pollution on hospital admissions with a focus on respiratory diseases: a time-series multi-city analysis. Environ Sci Pollut Res Int. 2019;26:16998–17009. 30929168 10.1007/s11356-019-04781-3 PMC6546668

[12] Kim H Kim H Lee JT. Spatial variation in lag structure in the short-term effects of air pollution on mortality in seven major South Korean cities, 2006-2013. Environ Int. 2019;125:595–605. 30765192 10.1016/j.envint.2018.09.004

[13] Mentz G Robins TG Batterman S. Acute respiratory symptoms associated with short term fluctuations in ambient pollutants among schoolchildren in Durban, South Africa. Environ Pollut. 2018;233:529–539. 29102883 10.1016/j.envpol.2017.10.108 PMC5764788

[14] Xu D Chen Y Wu L. Acute effects of ambient PM(2.5) on lung function among schoolchildren. Sci Rep. 2020;10:4061. 32132612 10.1038/s41598-020-61003-4 PMC7055357

[15] Chen C Li C Li Y. Short-term effects of ambient air pollution exposure on lung function: a longitudinal study among healthy primary school children in China. Sci Total Environ. 2018;645:1014–1020. 30248826 10.1016/j.scitotenv.2018.07.154

[16] Ratajczak A Badyda A Czechowski PO. Air pollution increases the incidence of upper respiratory tract symptoms among Polish children. J Clin Med. 2021;10:2150. 34065636 10.3390/jcm10102150 PMC8156299

[17] Naidoo RN Robins TG Batterman S. Ambient pollution and respiratory outcomes among schoolchildren in Durban, South Africa. SAJCH. 2013;7:127–134. 10.7196/sajch.598 PMC4346135 25741408

[18] Department of Environmental Affairs. The Second Generation Vaal Triangle Airshed Priority Area Air Quality Management Plan: Draft Baseline Assessment Report. Pretoria. 2019. Available at: https://saaqis.environment.gov.za/pagesfiles/VTAPA%20SECOND%20GENERATION%20AQMP_DRAFT%20BASELINE%20ASSESSMENT%20REPORT_PUBLIC%20COMMENT.pdf. Accessed April 27, 2022.

[19] Terblanche P. Vaal Triangle Air Pollution Health Study: Bibliography, summary of key findings and recommendations. Medical Research Council; 1998.

[20] Department of Environmental Affairs. Declaration of the Vaal Airshed Priority Area. 2006. Available at https://www.gov.za/sites/default/files/gcis_document/201409/28732b.pdf. Accessed April 27, 2022.

[21] Department of Environmental Affairs. The 2017 National Framework for Air Quality Management in the Republic of South Africa. National Environmental Management: Air Quality Act, 2004 (Act No. 39 of 2004), Government Gazette, No. 1144. Pretoria; 2018.

[22] Department of Environmental Affairs. National Ambient Air Quality Standards, NEM:AQA, Government Gazette, No 1210. Pretoria; 2009.

[23] Department of Environmental Affairs. National Ambient Air Quality Standard for Particulate Matter with Aerodynamic Diameter less than 2.5 micrometers, National Ambient Air Quality Standards, NEM:AQA, Government Gazette, No. 486. Pretoria; 2012.

[24] South African Weather Service. Vaal Triangle Air Quality Monitoring Network Monthly Report. Available at: https://saaqis.environment.gov.za/. Accessed April, 2022.

[25] Lewis TC Robins TG Dvonch JT. Air pollution-associated changes in lung function among asthmatic children in Detroit. Environ Health Perspect. 2005;113:1068–1075. 16079081 10.1289/ehp.7533 PMC1280351

[26] Graham BL Steenbruggen I Miller MR. Standardization of spirometry 2019 update. An Official American Thoracic Society and European Respiratory Society Technical Statement. Am J Respir Crit Care Med. 2019;200:e70–e88. 31613151 10.1164/rccm.201908-1590ST PMC6794117

[27] National Asthma Education and Prevention Program. Expert Panel Report 3: Guidelines for the Diagnosis and Management of Asthma. U.S. Department of Health and Human Services; National Institutes of Health; National Heart, Lung, and Blood Institute, Bethesda, MD; 2007.

[28] Persinger RL Poynter ME Ckless K. Molecular mechanisms of nitrogen dioxide induced epithelial injury in the lung. Mol Cell Biochem. 2002;234–235:71–80. 12162462

[29] Ranzi A Freni Sterrantino A Forastiere F. Asthmatic symptoms and air pollution: a panel study on children living in the Italian Po Valley. Geospat Health. 2015;10:366. 26618316 10.4081/gh.2015.366

[30] Prieto-Parra L Yohannessen K Brea C. Air pollution, PM2.5 composition, source factors, and respiratory symptoms in asthmatic and nonasthmatic children in Santiago, Chile. Environ Int. 2017;101:190–200. 28202226 10.1016/j.envint.2017.01.021

[31] Mentz G Robins TG Batterman S. Effect modifiers of lung function and daily air pollutant variability in a panel of schoolchildren. Thorax. 2019;74:1055–1062. 31534032 10.1136/thoraxjnl-2017-211458

[32] Watanabe M Noma H Kurai J. Association between pulmonary function and daily levels of sand dust particles assessed by light detection and ranging in schoolchildren in western Japan: a panel study. Allergol Int. 2016;65:56–61. 26666494 10.1016/j.alit.2015.07.005

[33] Hsu SC Chang JH Lee CL. Differential time-lag effects of ambient PM(2.5) and PM(2.5)-bound PAHs on asthma emergency department visits. Environ Sci Pollut Res Int. 2020;27:43117–43124. 32729038 10.1007/s11356-020-10243-y

[34] Hwang SL Lin YC Lin CM. Effects of fine particulate matter and its constituents on emergency room visits for asthma in southern Taiwan during 2008-2010: a population-based study. Environ Sci Pollut Res Int. 2017;24:15012–15021. 28488152 10.1007/s11356-017-9121-3

[35] Dales R Chen L Frescura AM. Acute effects of outdoor air pollution on forced expiratory volume in 1 s: a panel study of schoolchildren with asthma. Eur Respir J. 2009;34:316–323. 19251781 10.1183/09031936.00138908

[36] Kalandidi A Gratziou C Katsouyanni K. Air pollution and respiratory health of children: the PEACE panel study in Athens, Greece. Eur Respir Rev. 1998;52:117–124.

[37] Weinmayr G Romeo E De Sario M. Short-term effects of PM10 and NO2 on respiratory health among children with asthma or asthma-like symptoms: a systematic review and meta-analysis. Environ Health Perspect. 2010;118:449–457. 20064785 10.1289/ehp.0900844 PMC2854719

[38] World Health Organization (WHO). WHO global urban ambient air pollution database (update 2018, version 11). World Health Organization, Geneva. 2018a. Available at: https://www.who.int/data/gho/data/indicators/indicator-details/GHO/concentrations-of-fine-particulate-matter-(pm2-5). Accessed April 27, 2022.

[39] European Environment Agency. Air Quality Status 2021. Available at: https://www.eea.europa.eu/publications/air-quality-status-2021. Accessed April 27, 2022.

[40] US Environmental Protection Agency. Our Nation’s Air: Trends through. 2020. Available at: https://gispub.epa.gov/air/trendsreport/2021/#home. Accessed April 27, 2022.

[41] World Health Organization. WHO global air quality guidelines: particulate matter (PM2.5 and PM10), ozone, nitrogen dioxide, sulfur dioxide and carbon monoxide. 2021. Available at: https://apps.who.int/iris/handle/10665/345329. Accessed April 27, 2022. 34662007

