# Supplementary material for: Lagged acute respiratory outcomes among children related to ambient pollutant exposure in a high exposure setting in South Africa
Source: Environ Epidemiol. 2022 Nov 7;6(6):e228. doi: 10.1097/EE9.0000000000000228 (PMC9746739; doi:10.1097/EE9.0000000000000228)
Supplement: Supplementary file 1 [file ee9-6-e228-s001.docx]

Supplementary Material

Lagged acute respiratory outcomes among children related to ambient pollutant exposure in a high exposure setting in South Africa

Classification of Asthma, based on reported symptoms

“Any asthma” was defined as the presence of a positive response to the question on:

- Presence of above non-exercise symptoms more frequently than 3-12x per year
- Presence of symptoms with exercise more frequently than 3-12x per year
- Doctor diagnosed asthma
- Asthma medication

The classification of asthma severity was based on the following:

- Moderate-severe: “Any asthma” + symptoms shown below was present every day
- Mild Persistent: “Any asthma” + symptoms more than twice per week, but not daily
- Mild Intermittent: all other forms of “any asthma”

Persistent cough Y/N

| In the past 12 months, how often has your child had a cough that won’t go away? | 🞎_1_ Every day  🞎_2_ More than 2 times per week  🞎_3_ More than 1 time per month  🞎_4_ 3 to 12 times in the whole year  🞎_5_ 1 or 2 times in the whole year  🞎_6_ Never |
| --- | --- |

Wheezing frequency Y/N

| In the past 12 months, how often has your child had wheezing (a whistling sound from the chest) *without* a cold? | 🞎_1_ Every day  🞎_2_ More than 2 times per week  🞎_3_ More than 1 time per month  🞎_4_ 3 to 12 times in the whole year  🞎_5_ 1 or 2 times in the whole year  🞎_6_ Never |
| --- | --- |

Wheezing with breathlessness: Y/N

| In the past 12 months, how often has your child had an attack of wheezing that made it hard for him or her to breathe or catch his or her breath? | 🞎_1_ Every day  🞎_2_ More than 2 times per week  🞎_3_ More than 1 time per month  🞎_4_ 3 to12 times in the whole year  🞎_5_ 1 or 2 times in the whole year  🞎_6_ Never |
| --- | --- |

Tight chest Y/N

| In the past 12 months, how often has your child complained that his or her chest felt tight or heavy? | 🞎_1_ Every day  🞎_2_ More than 2 times per week  🞎_3_ More than 1 time per month  🞎_4_ 3 to 12 times in the whole year  🞎_5_ 1 or 2 times in the whole year  🞎_6_ Never |
| --- | --- |

Chest problems disturbing sleep

| In the past 12 months, how often has your child’s sleep been disturbed due to wheezing, coughing, chest tightness or shortness of breath? | 🞎_1_ Most nights  🞎_2_ More than 2 times per week  🞎_3_ More than 1 time per month  🞎_4_ 3 to 12 times in the whole year  🞎_5_ 1 or 2 times in the whole year  🞎_6_ Never |
| --- | --- |

Wheeze with exercise/running

| In the past 12 months, how often has your child wheezed with exercise or running or playing hard? | 🞎_1_ Every day  🞎_2_ More than 2 times per week  🞎_3_ More than 1 time per month  🞎_4_ 3 to12 times in the whole year  🞎_5_ 1 or 2 times in the whole year  🞎_6_ Never |
| --- | --- |

Cough with exercise/running

| In the past 12 months, how often has your child coughed with exercise or running or playing hard? | 🞎_1_ Every day  🞎_2_ More than 2 times per week  🞎_3_ More than 1 time per month  🞎_4_ 3 to 12 times in the whole year  🞎_5_ 1 or 2 times in the whole year  🞎_6_ Never |
| --- | --- |

Asthma medication

| In the past 12 months has your child taken any medications, nebulisers, or inhalers (pumps) prescribed by a doctor for any of chest conditions? | _1_ Yes  _2_ No |
| --- | --- |
